# Generalization of learned preferences covaries with behavioral flexibility in red junglefowl chicks

**DOI:** 10.1093/beheco/arz088

**Published:** 2019-07-13

**Authors:** Josefina Zidar, Alexandra C V Balogh, Olof Leimar, Hanne Løvlie

**Affiliations:** 1 Department of Physics, Chemistry and Biology, IFM Biology, Linköping University, Campus Valla, inköping, Sweden; 2 Department of Zoology, Stockholm University, Stockholm, Sweden

**Keywords:** animal cognition, animal personality, coping style, *Gallus gallus*, learning

## Abstract

The relationship between animal cognition and consistent among-individual behavioral differences (i.e., behavioral types, animal personality, or coping styles), has recently received increased research attention. Focus has mainly been on linking different behavioral types to performance in learning tasks. It has been suggested that behavioral differences could influence also how individuals use previously learnt information to generalize about new stimuli with similar properties. Nonetheless, this has rarely been empirically tested. Here, we therefore explore the possibility that individual variation in generalization is related to variation in behavioral types in red junglefowl chicks (*Gallus gallus*). We show that more behaviorally flexible chicks have a stronger preference for a novel stimulus that is intermediate between 2 learnt positive stimuli compared to more inflexible chicks. Thus, more flexible and inflexible chicks differ in how they generalize. Further, behavioral flexibility correlates with fearfulness, suggesting a coping style, supporting that variation in generalization is related to variation in behavioral types. How individuals generalize affects decision making and responses to novel situations or objects, and can thus have a broad influence on the life of an individual. Our results add to the growing body of evidence linking cognition to consistent behavioral differences.

## Teaser

Red junglefowl chicks generalize learned preferences differently depending on their behavioral type (aka personality). When an individual generalizes, previous experiences are used when responding to novel situations or objects. Variation in generalization can thus affect decision making and the action taken when responding to novel situations. Here, we show that more cognitively flexible chicks react more strongly to a novel stimulus in a generalization task than less flexible chicks do.

## INTRODUCTION

Generalization is a psychological mechanism where responses to novel stimuli are similar to responses towards previously experienced stimuli. It reduces the need to learn details of novel stimuli separately and renders decision making more efficient. Generalization is therefore an aspect of cognition that is essential in decision making and can affect fitness-related traits (Guilford and Dawkins 1991). Because generalization reduces the amount of information that an individual needs to process (Kelber 2018), how broadly an individual generalizes decides how much and how accurately that individual handles information about an object at hand. Generalization can therefore influence foraging decisions and mate choice, and this, in turn, can have effects on the evolution of, for example, prey warning coloration (e.g., Gamberale Stille et al. 2018) and sexual ornaments (ten Cate and Rowe 2007). Different aspects of generalization have been studied extensively during the last century (for review, see Ghirlanda and Enquist 2003) in a variety of species, such as pigeons (*Columba livia*, e.g., Klein and Rilling 1974; Guillette et al. 2017), rats (*Rattus norvegicus*, e.g., Brennan and Riccio 1972), and domestic fowl (*Gallus gallus domesticus,* e.g., Jones et al. 2001; Osorio et al. 2009).

It has long been known that a generalized response towards a novel stimulus can be stronger than responses to familiar stimuli, for example, when the novel stimulus is intermediate between familiar ones (e.g., Spence 1937). More recently, this stronger generalized response towards a novel stimulus was used to test how robust a response is towards a generalized stimulus compared to a learned stimulus, in other words, how fast the response towards the 2 stimuli declines when left unrewarded (Osorio et al. 2009). In that study, domestic fowl chicks learned to associate a red and a yellow color with rewards and were then presented to a novel, intermediate orange color. As expected by generalization theory (e.g., Spence 1937; Hanson 1959), the chicks showed a stronger generalized response (towards the novel color) than the learned response (towards the learned colors). Also, the generalized preference was more quickly lost than the learned preference, indicating that a generalized preference is less stable and more affected by new information through experience than a learned preference is. However, the generalized response was much stronger than predicted by generalization theory (Osorio et al. 2009). This exaggerated, even stronger than expected, preference for an intermediate color stimulus seems to be a robust generalization phenomenon and it has been shown along several color gradients (e.g., blue and green with an intermediate turquoise, Jones et al. 2001, and red and yellow with an intermediate orange, Osorio et al. 2009). It is therefore unlikely to be an effect of an innate preference for any specific color. Nor is it novelty per see that gives rise to the strong response, since individuals do not respond as strongly when presented with a novel color outside the region of color space spanned by the 2 learned colors (Jones et al. 2001; Osorio et al. 2009). A suggested explanation for the strong generalized response is that a novel stimulus gives rise to a higher uncertainty about the consequences of responding to it than a familiar stimulus would, and that this increases the attention paid to it (Osorio et al. 2009). Individual variation in generalization is theoretically predicted to be related to the behavioral type of individuals (Sih and Del Giudice 2012). Behavioral types are described as consistent behavioral differences among individuals, also known as animal personality or coping styles (Koolhaas et al. 1999; Dall et al. 2004; Sih et al. 2004).

Behavioral types can differ in cognitive traits (Sih and Del Giudice 2012; Griffin et al. 2015; Dougherty and Guilliette 2018). Research has primarily focused on aspects of learning (e.g., Light et al. 2011; Titulaer et al. 2012; Zidar et al. 2018) including problem solving (Cole et al. 2011; Zandberg et al. 2017). For example, bolder and more active bank voles (*Myodes glareolus*) learned a discrimination task more quickly but were slower in reversal learning than shyer and less active individuals (Mazza et al. 2018), fast-exploring great tits (*Parus major*) more often chose novel foraging options and are perhaps more susceptible to new information than slow explorers, who rely more on already established knowledge (Smit and van Oers 2019), and coping style has been shown to influence the speed with which hens learn to associate a color with a reward (de Haas et al. 2017). Generalization has also been found to be linked to personality, with fast-exploring pigeons generalizing more narrowly than slow explorers (Guillette et al. 2017). However, a possible link between coping style and generalization has not previously been shown.

According to the coping style literature, more proactive behavioral types are less susceptible to stress and tend to form and stick to established routines, compared to more easily stressed and fearful, reactive individuals who are more behaviorally flexible (Koolhaas et al. 1999; Coppens et al. 2010). Proactive and reactive individuals are hypothesized to consider and evaluate novel stimuli differently (Sih and Del Giudice 2012). If reactive individuals pay more attention to changes in their environment and thus are more cognitively flexible, one should expect that they generalize novel stimuli differently from proactive individuals. The observed exaggerated generalized response towards a novel intermediate color (Osorio et al. 2009) and the rapid decline of responses when left unrewarded indicate that generalization towards an intermediate stimulus might be affected by flexibility and attention. Based on the predicted relationship between generalization and behavioral types, and that it remains untested how individual variation explains variation in strength of generalization preference, we exposed red junglefowl (*Gallus gallus*) chicks (an established model for personality and cognition research, Garnham and Løvlie 2018), to a generalization task (inspired by Osorio et al. 2009) followed by a series of personality assays. We hypothesize that more cognitively flexible, reactive individuals would respond more strongly to a novel intermediate stimulus than less flexible, more proactive individuals.

## METHODS

### Animals and housing

We used a captive population of red junglefowl at Linköping University, Sweden. This study population has been pedigree-bred since 2011, and not subject to any intentional directional selection (see Zidar et al. 2018 for further details on the background of the population, and Sorato et al. 2018 for further details on the family structure of the population). We used 2 batches of red junglefowl chicks (*n* = 67; *n*_males_ = 27; *n*_females_ = 37, *n*_unknown sex_ = 5) from 18 families, March–April 2013. All individuals were artificially incubated and reared in groups without their mothers, thereby reducing maternal influences on development of personality and cognitive performance (Stamps and Groothuis 2010). Chicks were kept in same-age, mixed-sex groups, in 3 cages ranging from 0.5 to 3 m^2^ (cages increasing in size as the chicks grew larger). All chicks had access to dust baths, perches, commercial poultry food and water, ad libitum. Room temperature was kept around 24–27 °C, and the first week chicks had access to heat lamps. During testing, chicks were moved to a lab room and housed in experimental cages. These cages were like their home cages with access to perches, wood shavings (enabling dust bathing), and ad libitum access to commercial poultry food and water. Room temperature was around 23–26 °C, and chicks had access to dark brooders (which supply additional heat) in their cages the first week. All individuals were marked with wing-tags to facilitate recognition. After habituation to human handling and the test arena, chicks were singly exposed to discrimination (including a measure of behavioral flexibility) and generalization tasks followed by 3 personality assays. Training and testing were carried out between 8 and 18 local time (lights were on 7–19). At 8 weeks of age and after the experimental period, chicks were moved to the facility were adult birds at Linköping University are kept. Birds were here housed in same-sex groups and had access to an indoor and an outdoor area (3 + 3 m^2^).

The study was approved by Linköping Ethical committee (ethical permit number 122-10) and followed legal requirements in Sweden.

### Stimulus construction

The stimulus dimension we used was along the color gradient ranging from red to yellow (Supplementary Figure S1), following Osorio, Jones, et al. 1999a; Osorio, Vorobyev, et al. 1999b and Jones et al. 2001. The colors were chosen according to their photoreceptor excitation effects (Supplementary Figure S2), in other words, constructed by taking the color vision of domestic chicken into account. We used the spectral sensitivities of the chicken retina (see Figure 2 in Jones et al. 2001) to determine photoreceptor excitations as values in a 2-dimensional color space, based on the relative excitation of 3 types of photoreceptors, corresponding to short, medium, and long wavelengths (Supplementary Figure S2, see also Osorio, Jones, et al. 1999a). In addition, the excitation of the double cones in the eye of domestic chicks was used as a measure of stimulus lightness. Birds also have an additional type of receptor with sensitivity for ultraviolet light, but we assumed that this sensitivity could be ignored in the light environment of the experimental setting. We used daylight lamps emitting no or very little UV wavelengths, and we also used a filter to remove any possible UV-components. For the analysis of the photoreceptor excitations in the chicken retina, (see Supplementary Information). The color stimuli were presented as patterns on the surface of printed paper cones and consisted of rectangular gray and colored tiles (Supplementary Figure S1, see also Osorio, Jones, et al. 1999a). The wavelength reflectance spectra of color prints were measured with a light spectrometer (Ocean Optics, Dunedin, FL). The colors used were red, yellow, and orange, with the latter being intermediate between the first 2 in color space (Supplementary Figure S2). Three similar shades of the same color intermingled with gray were used on the same stimulus cone to reduce the possibility of differing contrasts between the gray and colored tiles on differently colored cones. The 3 shades were assigned randomly to tiles within the pattern (Suplementary Figure S1). Additionally, we made cones consisting of tiles in different shades of gray (Supplementary Figure S1). These cones should not influence generalization and thus functioned as a control.

### Experimental setup

#### Discrimination training and generalization test

Training started when chicks where 14 days old and the setup was inspired by the design by Osorio et al. (2009). Chicks were handled and gently familiarized with the test arena (28 × 18 × 37 cm), as well as with temporary isolation from their pen mates already 1-day post hatching (Zidar, Balogh, et al. 2017; Zidar, Sorato, et al. 2017; Sorato et al. 2018; Zidar et al. 2018). Red and yellow cones were filled with a mixture of chopped mealworms, chicken crumbs, and water, and were along with the unrewarded empty gray cones placed on the arena floor (46 × 37 cm, Supplementary Figure S3). Chicks were trained with 6 cones (2 red, 2 yellow, and 2 gray), for 6 min, 3 times daily for 2 consecutive days. Emptied cones were immediately refilled. At day 3 of training, a seventh and last training session took place. One hour thereafter, a generalization test was performed, and consisted of 2 consecutive trials (1 h apart). In these trials, chicks were given 9 cones: 3 gray, 3 either red or yellow (balanced between the colors), and 3 novel, intermediate orange cones. Generalization was tested in extinction (i.e., all cones were unrewarded), to reduce the risk of further learning and chicks choosing colors based on whether they were rewarded or not. To measure the chicks’ color preferences (including its change over time in extinction) in the generalization test, the color of each chick’s ten first chosen cones in each trial was recorded. A cone was considered chosen if the chick physically interacted with it by pecks (or kicks, a strategy of chicks to extract the expected reward). Repeated pecks on the same cone were not counted as a new choice, unless another cone had been pecked on in between. The choice sequence is referred to as “peck” 1–10 for each trial in the statistical analysis.

When chicks were between 3 and 6 days old, they were exposed to discrimination and reversal learning tests (as part of another study, Zidar, Balogh, et al. 2017; Zidar, Sorato, et al. 2017; Sorato et al. 2018; Zidar et al. 2018). Behavioral flexibility was measured in the transition between these 2 tests and was used in this study. At 4 and 6 weeks of age, all chicks were tested in a battery of personality assays including a novel arena, a novel object, and a tonic immobility test (sensu Zidar, Balogh, et al. 2017; Zidar, Sorato, et al. 2017; Zidar et al. 2018). Consistency was confirmed for the behaviors recorded in these personality assays (see Zidar, Balogh, et al. 2017).

#### Novel arena test

An arena (76 × 114 cm) decorated with familiar food and water containers, novel to the chicks, was used to measure variation in exploration and fearfulness (Forkman et al. 2007; Réale et al. 2007). We changed location of these containers between test occasions to retain the novelty of the arena. Further, and for the same reason, we changed the substrate used: at 4 weeks of age, the substrate was wood shavings, while in the repeated test at 6 weeks of age, we instead used shredded cardboard paper. To prevent chicks from escaping, we placed a metal grid over the test arena. To measure how the birds used the arena, we divided the arena into 6 equally sized (imagined) sections. We placed the chick in the arena and used instantaneous sampling every 10 s for the 10 min to score their behaviors. The behaviors were observed directly via video cameras connected to a screen. Behaviors scored were “Latency to move” (i.e., latency until the bird started moving, measured in seconds since the test started), “Latency to visit all areas of the arena” (i.e., latency until the bird had visited all 6 imagined sections of the arena, measured in seconds since the test started), “Locomotion” (i.e., frequency of locomotion; walking, running), “Foraging” (i.e., frequency of time spent with its head down close to the ground either pecking at the floor or scratching the surface with its feet), “Vigilance” (i.e., frequency of time spent standing or walking with its eyes open and head high above shoulder-height), and “Number of escape attempts” (i.e., total number of times the chick tried to leave the arena).

#### Exposure to a novel object

A plush toy (ca 15 cm with *ca* 2 cm large eyes) that the chicks had never seen before, was used to measure variation in boldness and exploration (Réale et al. 2007). To reduce confounding effects of a novel environment when measuring the response to the novel object (Réale et al. 2007), we used the same arena as the chicks had familiarized themselves with in the novel arena test. Directly following the novel arena test, we placed the plush toy in the arena (while the light was switched off) as far away from the chick as possible. The same behaviors as described for the novel arena test, was scored during 10 min.

#### Tonic immobility

We scored the birds’ fear response in a tonic immobility test (Forkman et al. 2007). To induce a chick into tonic immobility it was placed on its back in a V-shaped wooden stand (20 × 10 cm). The observer held the bird down and limited the bird’s vision by placing a hand with a light pressure on the chest and by holding a hand over its eyes. The pressure was slowly released after 15 s and latency (in seconds) until the chick moved its head (“Latency to move TI”), was recorded. If a chick did not stay on its back for 3 s or more the procedure was repeated a maximum of 3 times. If we were unable to induce the chick into tonic immobility it received a score of 0 s. On the other hand, if a chick stayed immobile more than 10 min it received a score of 600 s. We used the same observer for all birds and the observer was unaware of the birds’ scores in the other tests.

#### Behavioral flexibility

To measure behavioral flexibility, all birds were initially taught to discriminate between 2 differently colored bowls (1 blue and 1 green), of which one contained a reward (Zidar, Balogh, et al. 2017; Zidar, Sorato, et al. 2017; Sorato et al. 2018; Zidar et al. 2018). Once a chick made 5 correct choices in a row, the association between color and reward was considered to be formed. We have previously shown that with a criterion of 5 correct choices in a row, the chance of putative learners being false positives is low (Sorato et al. 2018). After a chick had reached our criteria, the reward was instead placed in the previously unrewarded bowl. Variation in behavioral flexibility was measured by recording the latency for a chick to explore this bowl (called “reversal latency”). This measure is related to another measure of behavioral flexibility in our population (Zidar et al. 2017).

### Statistical analyses

To reduce the number of behavioral variables obtained in the novel arena, novel object, and tonic immobility tests (both obtained latencies and frequencies), we used principal component analysis (PCA). We used the mean response of behavioral measures obtained at 4 and 6 weeks of age. This resulted in 3 principal components: “PC1_active,” “PC2_nervous,” and “PC3_fearful” (Table 1).

**Table 1:** Principal component analysis of behavioral responses of red junglefowl chicks from personality assays

	“Active” (PC1)	“Nervous” (PC2)	“Fearful” (PC3)
Activity (NA)	**0.50**	−0.20	0.05
Vigilance (NA)	**0.49**	0.06	−0.09
Latency to move (NA)	−**0.37**	0.27	−0,12
Latency to explore all areas (NA)	−**0.46**	**0.32**	−0.18
Vigilance (NO)	**0.33**	**0.56**	−0.23
Number of escape attempts (NO)	0.22	**0.63**	0.00
Latency to move head (TI)	−0.03	0.25	**0.94**
Eigenvalues	2.75	1.23	1.00
Variance explained (%)	39.29	17.63	14.23

Eigenvalues and variance explained by components are presented. Values in bold have values ± > 0.30.

NA, Novel arena; NO, Novel object; TI, Tonic immobility. PC1 is interpreted as describing more vs. less active and explorative individuals. PC2 is interpreted as describing more vs. less nervous individuals. PC3 is interpreted as describing more vs. less fearful individuals.

In the generalization test, orange (novel cue), red/yellow (previously rewarded), and gray (previously unrewarded), were present simultaneously, and the relative preference for orange versus gray, and red/yellow versus gray, was used as a bivariate response (Table 2). In order to statistically estimate bivariate preferences for orange and red/yellow, we performed a Bayesian analysis, using the “MCMCglmm” package (see Supplementary Information for details).

**Table 2:** Variation in preference for the novel, generalized cue, and previously learnt cues in red junglefowl chicks

Preference	Fixed effect	Estimate	95% CI	*P*_MCMC_
Novel cue (orange)				
	Intercept	1.65	(1.20, 2.05)	**< 0.001**
	Peck number (1–10)	−0.07	(−0.12, −0.01)	**0.03**
	Trial (first vs. second)	−0.77	(−1.10, −0.40)	**< 0.001**
	Behavioral flexibility	0.17	(0.03, 0.32)	**0.01**
Learned cue (red/yellow)				
	Intercept	0.37	(−0.11, 0.81)	0.10
	Peck number (1–10)	−0.004	(−0.07, 0.06)	0.90
	Trial (first vs. second)	−0.31	(−0.67, 0.09)	0.11
	Behavioral flexibility	0.07	(−0.07, 0.22)	0.35

Estimates and Bayesian confidence intervals from fitting a Bayesian generalized mixed model given on the latent variables scales, which were “log(Pr [orange]/Pr [gray])” and “log(Pr [red or yellow]/Pr [gray]),” in other words describing preference for the novel (orange) and previously rewarded cues (red/yellow) over the unrewarded cue (gray). In this final model, “Trial” was a 2-level factor (first vs. second), and the covariate behavioral flexibility (“log(1/reversal latency)”) was centered (see Supplementary Information, Statistical analysis). Thus, an intercept of 0 means no preference for the color compared to gray, at the start of the first trial and for an average value of the behavioral covariate. Significant MCMC *P* values are shown in bold. See Supplementary Information for specification of prior and sampling parameters.

To investigate links between behavioral variation and generalization, we used generalized linear mixed models of the chicks’ choices. To select which variables to include in a final model, we first fitted logistic regressions (using “glmer” in the R package lme4). Each regression had the response variable “choosing orange” (yes/no), the fixed effects within-trial choice number “peck” (1–10) and “trial” (first vs. second), and the individual chick as random effect. To this model, we added 1 behavioral covariate at the time (i.e., “PC1_active,” “PC2_nervous,” “PC3_fearful” in Table 1, and “behavioral flexibility,” measured as the log of 1/“reversal latency,” to obtain normality). We used Akaike Information Criterion and *P* values (significant at α = 0.05 level) to determine which variables improved model fit. Only “behavioral flexibility” improved model fit, and was thus included in the final model. In addition, we examined whether the “color cue trained on” (i.e., the red, or yellow presented during testing), “family” (sib group), or “sex” added as a fixed effect to the final model, improved the model fit, but neither did.

In the final model, using the MCMCglmm package (see Supplementary Information), the choice of color by a chick (orange, red/yellow, or gray) was used as response variable. As explained in Supplementary Information, the model fitted so-called latent-variable responses that can be interpreted as the preference of chicks for the novel color (orange) and the learned colors (red/yellow), relative to gray (Table 2). We used within-trial choice number “peck” (1–10), “trial” (first vs. second), and “behavioral flexibility” as fixed effects, and chick “identity” as random effect. Because the generalization test was performed in extinction (all stimuli were unrewarded), the within-trial choice number “peck” and “trial” needed to be included in the analysis to estimate preferences at the start of the generalization test, before the effects of extinction became noticeable. In order to further illustrate how “behavioral flexibility” related to preference for the novel color cue, we performed a linear regression of the preference for orange (measured as the proportion of orange choices in trial 1), on “behavioral flexibility.” To explore the inter-relationship between “behavioral flexibility” and personality (“PC1_active,” “PC2_nervous,” “PC3_fearful”), we performed linear regressions.

## RESULTS

The PCA proposed 3 principal components, which we interpret as describing individuals that were more or less active (PC1), more or less nervous (PC2), and more or less fearful (PC3, Table 1).

**Figure 1:**
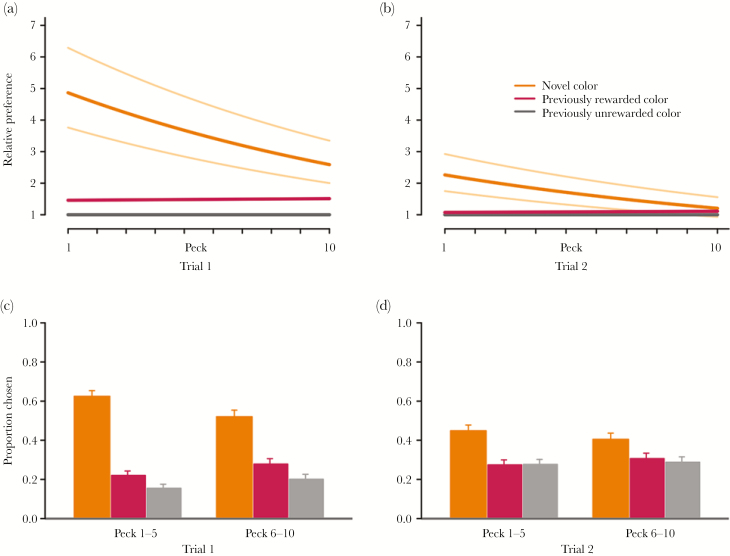
Generalization to a novel color by red junglefowl chicks. (a,b) Predictions from Bayesian MCMC model fitting, 10 first pecks (i.e., chosen cones), in each trial. The bold orange (upper line) and red (lower line) curves show the model estimated preference for the novel (orange) and previously rewarded (red and yellow) stimuli for chicks with average value of behavioral flexibility. The thinner, light orange curves show the model estimated preference for the novel color for chicks with behavioral flexibility 1 SD above (top curve) and below (bottom curve) the mean. (c,d) Proportions of different colors chosen (orange = left, red = middle, gray = right column), here visualized for the 1–5 and 6–10 pecks in each trial.

Chicks initially strongly preferred the novel color cue (orange), over a previously unrewarded color cue (gray) and also over the previously rewarded colors (red/yellow; Figure 1, Table 2, Bayesian 99.9% confidence interval (CI) for novel versus learnt cue intercept difference: 0.62–1.99). The preference for the novel color cue decreased with experience within and between trials (Figure 1, Table 2).

**Figure 2:**
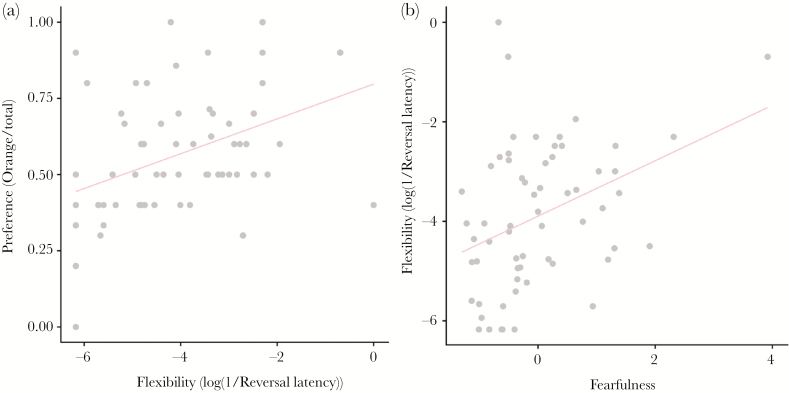
Relationship between generalization to the novel color orange and responses in behavioral assays by red junglefowl chicks. (a) Regression of the preference for orange, measured as the number of pecks on orange divided by the total number of pecks, on flexibility, and (b) flexibility on fearfulness.

Individual chicks differed in preference strength for the color cues, with standard deviations of random effects estimated around 0.5 (novel color cue: 95% CI: 0.35–0.69; previously rewarded colors: 95% CI: 0.32–0.62). Chicks that were measured to be more behaviorally flexible showed a stronger preference for the novel color than more inflexible individuals did (*t* = −3.51, *P* = 0.001, Figures 1 and 2a, Table 2). Behavioral flexibility did not relate to activity (PC1, *t* = 1.01, *P* = 0.27) or nervousness (PC2, *t* = 1.27, *P* = 0.21). However, behaviorally more flexible chicks were more fearful (PC3, *t* = −3.10, *P* = 0.003, Figure 2b).

## DISCUSSION

In the red junglefowl, we confirm that chicks strongly prefer a novel stimulus intermediate between 2 previously rewarded color stimuli, and that this preference decrease with time in the unrewarded generalization trials. More importantly, we explored this further, and show that there is individual variation in this preference, and that there is a positive correlation between the preference for the novel color stimulus and behavioral flexibility. Behavioral flexibility is an important aspect of coping styles (a.k.a. personality), in which individuals with a proactive behavioral type are less flexible and reactive individuals are more flexible (Koolhaas et al. 1999). Fearfulness is also an aspect of coping styles (Koolhaas et al. 1999) and because behavioral flexibility and fearfulness were positively correlated in our test birds, we interpret these variables as describing a proactive-reactive gradient, where flexibility and fearfulness describe more reactive individuals (Koolhaas et al. 1999). Therefore, our results suggest that generalization of learned information differs among individuals with different behavioral types, linking variation in animal cognition, and animal personality.

Our results suggest that chicks that can be described as more reactive generalize differently from more proactive chicks, by showing a higher preference for a novel color in our generalization test, compared to proactive chicks. The difference in response fits predictions that individuals of different behavioral types differ in aspects of cognition (Sih and Del Giudice 2012; Dougherty and Guilliette 2018). Speed-accuracy trade-offs have been hypothesized to influence the link between cognition and behavioral types (Koolhaas et al. 1999; Coppens et al. 2010; Sih and Del Giudice 2012). Proactive individuals are predicted to choose speed over accuracy, and therefore to spend less time to evaluate and sample new options, while reactive individuals will choose accuracy over speed and take their time evaluating several options (Sih and Del Guidice 2012). Additionally, information storing slows down decision making. Proactive individuals are therefore predicted to store less information than reactive ones (Sih and Del Giudice 2012). Reactive chicks may be more prone to interrupt an established routine, or perhaps less prone to establish a routine from the start, than proactive chicks (Koolhaas et al. 1999; Coppens et al. 2010; Sih and Del Giudice 2012). Proactive individuals thus may have a weaker preference for novel stimuli because they are more bound to their routine of focusing on previously rewarded stimuli and also less willing to spend time evaluating a novel color cue. One potential explanation of our result, that different levels of generalization are linked to differences in behavioral flexibility, is differences in attention towards novel stimuli. Our results are not directly comparable to previous work (Guillette et al. 2017) on the relation between generalization and personality. Guillette et al. (2017) showed that fast-exploring pigeons generalize more narrowly than slow-exploring pigeons. This might indicate that fast explorers judge stimuli more accurately than slow explorers. In general, the preference for a generalized stimulus, and therefore, the shape of such a gradient, might vary with time during a process of extinction. Individuals who are more attentive might have a higher preference for a generalized stimulus at the beginning of a generalization test (or a series of tests) but lose interest faster than individuals who pay less attention to new information. This would support the idea of Osorio et al. (2009) that attention could be an explanation for the elevated response towards a novel intermediate stimulus, but warrants further investigation. Variation in attention acting as a link between variation in behavioral types and generalization (and other aspects of cognition) is also consistent with theoretical predictions suggesting that reactive individuals are more attentive to new information (Koolhaas et al. 1999, Coppens et al. 2010; Sih and Del Giudice 2012).

It is well established that cognitive and personality traits can influence life-history variation and fitness in the wild (Morand-Ferron et al. 2016). For instance, it has been shown that personality in great tits is related to their foraging decisions when presented with unfamiliar resources compared to familiar ones (Smit and van Oers 2019). Concerning our results here, differential use of resources is one possible consequence of differences in generalization between individuals, because generalization affects judgment of food items during foraging. Reactive individuals might then more readily explore and make use of novel types of food. If such variation in resource use is associated with personality, it could affect the evolution of life histories (Wolf and Weissing 2012). In addition, individual variation in generalization over the appearances of food items can influence aspects of the evolution of prey warning coloration (Balogh and Leimar 2005). Individual variation in generalization can thus have broad consequences both for the individual, and for broader ecological and evolutionary concepts.

Taken together, our results demonstrate that variation in behavioral types, and mainly in behavioral flexibility, explains variation in generalization in red junglefowl chicks. Our work expands the growing body of literature showing a relationship between cognition and consistent behavioral differences among individuals and is to our knowledge the first study to explore how generalization of a learned preference when presented with a novel color cue covaries with behavioral type and behavioral flexibility. Future work should explore how general this pattern is across species, the consequences it has for the individual, and the ecological and evolutionary implications of that behavioral types generalize differentially, to overall improve our understanding of the consequences of this observed covariation.

## FUNDING

A.B. was funded by The Swedish Research Council and Helge Ax:son Johnson’s foundation. H.L. was supported by the program “Future research leaders” at Linköping University and the Swedish Research council FORMAS. The work was carried out within the collaborative network of the Centre of Excellence in Animal Welfare Science.

## Supplementary Material

arz088_Suppl_Supplementary_Materials
