# Supplementary material for: Generalization of learned preferences covaries with behavioral flexibility in red junglefowl chicks
Source: Behav Ecol. 2019 Jul 13;30(5):1375–81. doi: 10.1093/beheco/arz088 (PMC6765379; doi:10.1093/beheco/arz088)

Supplementary material for:

Generalization of learned preferences covaries with behavioural flexibility in red junglefowl chicks.

Josefina Zidar, Alexandra C.V. Balogh, Olof Leimar & Hanne Løvlie.

This file contains:

- **Supplementary methods**
- **Supplementary statistical analyses**
- **Supplementary references**
- **Supplementary figures**
    - Figure S1 Stimuli used in generalization task for red junglefowl chicks.
    - Figure S2 Photoreceptor simulations in color space.
    - Figure S3 A photo of a red junglefowl chick in a training arena.

Supplementary methods

Construction of stimuli

The analysis of color stimuli, taking the photoreceptor excitations effects of colors on the chicken retina into account, followed that of Osorio et al. (1999a, b). It was conducted according to the following: we used the spectral sensitivities of the retina $P_s(\lambda)$, $P_m(\lambda)$, $P_l(\lambda)$ and $P_d(\lambda)$ for the different photoreceptor types (Jones et al. 2001), where λ is the wavelength of the light and *s*, *m*, *l* and *d* represent short, medium, long and double cone receptor types. We normalized these functions between λ = 300 nm and λ = 700 nm according to

$$\int_{300}^{700} P_i(\lambda)d\lambda = 1. \quad (1)$$

The spectral distribution of irradiance, i.e. the light environment, was also normalized as

$$\int_{300}^{700} I(\lambda)d\lambda = 1. \quad (2)$$

The measured reflectance of the stimuli surface is a function of wavelength, $S_k(\lambda)$, for one or more stimuli *k*. We defined a white standard reflectance S_{white}= 1 to define the achromatic central point in the colour (see fig. S2). In order to calculate the positions of the stimuli colours in colour space, the relative photoreceptor stimulations p_s, p_m and p_l were calculated by determining the quantum catch of receptor type *i*,

$$Q_i(S)\int_{300}^{700} P_i(\lambda)S(\lambda)I(\lambda)d\lambda, \quad (3)$$

and from this, the relative quantum catch of receptor type *i,*

$$q_i = \frac{Q_i(S_k)}{Q_i(S|\ |\ )}, \quad (4)$$

was used to attain the relative photoreceptor stimulations,

$$p_i = \frac{q_i}{q_s+q_m+q_l}. \quad (5)$$

The relative photoreceptor stimulations were then used to calculate the positions in the colour triangle according to

$$x = \frac{1}{\sqrt{2}}(p_l - p_s)$$
$$y = \sqrt{\frac{2}{3}}\left(p_m - \frac{1}{2}(p_s + p_l)\right). \quad (6)$$

Note that $p_s + p_m + p_l = 1$, and that for $p_s = p_m = p_l = 1/3$, we get the achromatic point $x = 0$, $y = 0$ in colour space, see fig. S1).

To control the lightness of stimuli k, we determined it as the relative stimulation of the double cone receptors S_k and a perfectly reflecting surface, S_{white}. With the quantum catch of the double cones

$$Q_d(S) = \int_{300}^{700} P_d(\lambda)S(\lambda)I(\lambda)d\lambda, \quad (7)$$

the lightness of stimulus surface k is

$$B(S_k) = Q_d. \quad (8)$$

Statistical analysis

By using the Bayesian MCMCglmm package (Hadfield 2010, 2018), we were able to analyse the preference for both orange and red/yellow in the same statistical model. Our model fitting follows the example in section 5.2 of Hadfield (2018). The response variable in our model is the choice by a chick, either orange, red/yellow, or gray. The model fits the probabilities of these choices using so-called latent variables, as follows. The probability of choosing the unrewarded gray represents a base level, and we can use a bivariate response in the form of two latent variables. The latent variable l_1 is the log of the ratio of the probabilities of choosing orange and choosing gray, and l_2 is the log of the ratio of probabilities of choosing a learned colour (red or yellow) and choosing gray. We can regard these latent variables as preferences for orange and for a red/yellow. Using MCMCglmm, the analysis is the fitting of a Bayesian MCMC mixed model for the latent bivariate response (l_1, l_2). The actual response variable is the choice (orange, red/yellow, gray) of an individual. We used the 10 first choices (variable Peck) in Trial 1 and the 10 first choices in Trial 2 as data points, and we included the individual as a random effect. Because there is a bivariate response, the variance components will be two-dimensional variance-covariance matrices.

The (residual) variance component corresponding to a single choice cannot be estimated, because there is no replication at this level, and it was set following a recommendation by the author of the package (Hadfield, 2018). In addition to the residual, we included a variance component corresponding to the individual chick. Our prior was as follows

```
IJ <- (1/3) * (diag(2) + matrix(1, 2, 2))
prior1 = list(R = list(V = IJ, fix = 1),
        G = list(G1 = list(V = diag(2), nu = 2)))
```

The prior for the residual variance component is the same as the one used on p. 97 of Hadfield (2018). The idea behind it is that log(Pr (Choice = gray)) contributes to all variances and covariances, giving the term matrix (1, 2, 2) and, in addition, log(Pr (Choice = orange)) and log(Pr (Choice = learned colour)) each contribute to the variances of l_1 and l_2, giving the term diag(2). For the random effect (G1), we follow Hadfield (2018) in using a value of nu equal to the dimension of the random effect variance-covariance matrix (although smaller values of nu give similar conclusions). For the MCMC sampling, we used 10000 burn-in iterations, followed by 210000 iterations. Using a thinning interval of 200, we then got 1000 MCMC

samples. Examination of diagnostic plots and the effective number of samples indicated that the model fitting worked well. The final model was essentially as follows

```
fm <- MCMCglmm(Choice ~ trait + trait:Trial + trait:Peck +
        trait:clRl - 1,
        random = ~ us(trait):Chick,
        rcov = ~ us(trait):units,
        family = "categorical", prior = prior1)
```

where ‘Peck’ enumerates the sequence of choices by a chick and ‘clTl’ is the centred log(reversal latency) covariate. In addition to the results reported in the main text (Table 1), the estimates (Bayesian 95% confidence interval) for the chick-level variance component, expressed on the standard deviation scale, was 0.51 (0.35-0.69) for the preference for orange, and 0.47 (0.32-0.62) for the preference for red/yellow.

Adding the particular training color (yellow or red) a chick had been tested on to the model yielded a statistically non-significant effect and did not improve the model fit (not shown).

The data file and R-script for the supplementary analysis are available as supplementary files to the accepted article (‘chick_peck_data1.txt’ and ‘ana_chick_peck.R’).

References

Hadfield JD. 2010. MCMC methods for multi–response generalised linear mixed models: The MCMCglmm R Package. J Stat Software 33:1–22.

Hadfield JD. 2018. MCMCglmm course notes; available at: https://cran.r-project.org/web/packages/MCMCglmm/vignettes/CourseNotes.pdf

Jones CD, Osorio D, Baddeley RJ. 2001. Colour categorization by domestic chicks. Proc Roy Soc B. 268:2077–2084.

Osorio D, Jones CD, Vorobyev M. 1999a. Accurate memory for colour but not pattern contrast in chicks. Curr Biol. 9:199–202.

Osorio D, Vorobyev M, Jones CD. 1999b. Colour vision of domestic chicks. J Exp Biol. 202:2951–2959.

Supplementary figures

Figure S1. Stimuli used in generalization task for red junglefowl chicks. Red, orange, yellow and gray, respectively, presented from left to right. The yellow stimulus (third from the left) appears green to the human eye.

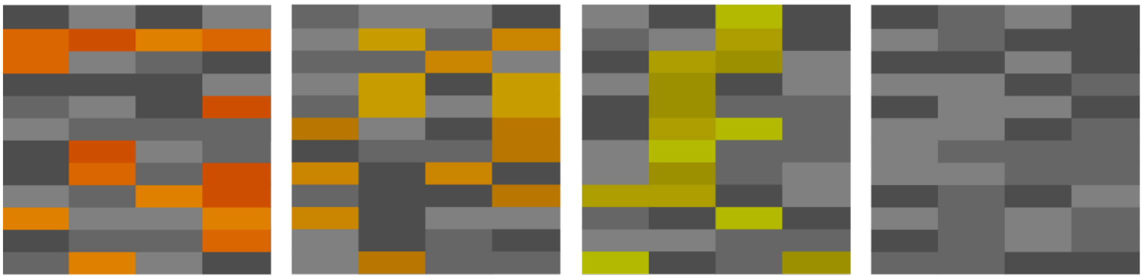

Figure S2. Photoreceptor simulations in color space. Photoreceptor stimulations in color space for three shades of each of the colors red (lower right), orange (middle right), yellow (upper right) and gray (middle of figure).

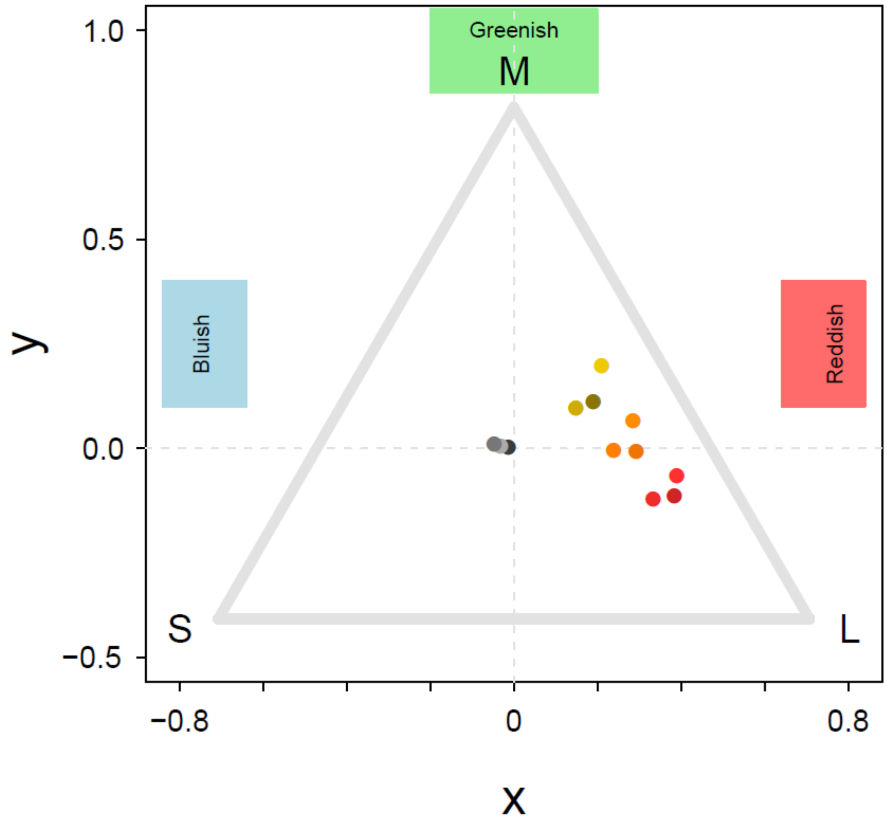

Figure S3. A photo of a red junglefowl chick in the training arena. Cones (red, yellow, gray) are constructed from stimuli shown in Figure S1. Note that the number of cones in the arena in this photo differs from the actual training setup (please see the methods in the main paper).

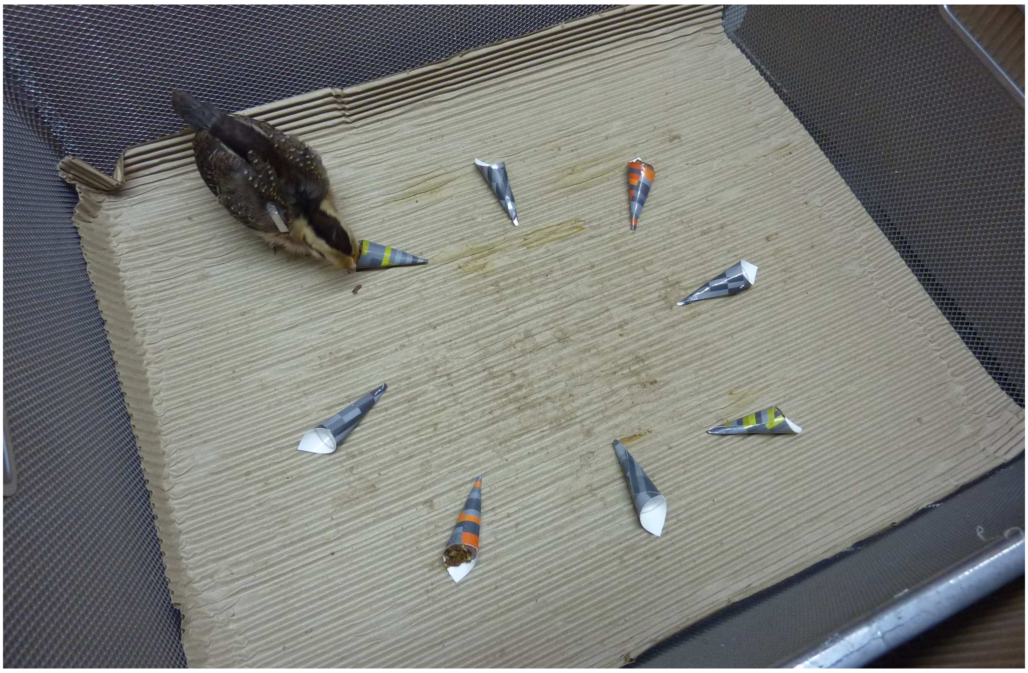

Supplement: arz088_Suppl_Supplementary_Materials [file arz088_suppl_supplementary_materials.pdf]
